# “I lost my happiness, I felt half dead and half alive” - a qualitative study of the long-term aftermath of obstetric near-miss in the urban district of Zanzibar, Tanzania

**DOI:** 10.1186/s12884-020-03261-8

**Published:** 2020-10-07

**Authors:** Tanneke Herklots, Suhaila Salum Yussuf, Khairat Said Mbarouk, Molly O’Meara, Emma Carson, Sebastiaan Beschoor Plug, Fleur van Acht, Pleun Terpstra, Deja Prebevšek, Arie Franx, Tarek Meguid, Benoit Jacod

**Affiliations:** 1 Department of Obstetrics & Gynaecology, Erasmus Medical Center, Rotterdam, the Netherlands; 2 Department of Obstetrics & Gynaecology, Mnazi Mmoja Hospital, Zanzibar, United Republic of Tanzania; 3 School of Global Health, University of Copenhagen, Copenhagen, Denmark; 4 Faculty of Humanities, University Utrecht, Utrecht, the Netherlands; 5 Department of Technology, Policy and Management, Technical University Delft, Delft, the Netherlands; 6 Faculty of Medicine, University Utrecht, Utrecht, the Netherlands; 7 Faculty of Medicine, University of Maastricht, Maastricht, the Netherlands; 8 Village Health Centre, Kigutu, Burundi; 9 Department of Obstetrics & Gynaecology, Onze Lieve Vrouwe Gasthuis Hospital, Amsterdam, the Netherlands

**Keywords:** maternal morbidity, obstetric complications, postpartum period, long-term impact, low-income setting, value-based health care

## Abstract

**Background:**

This study aims to explore the stories of three women from Zanzibar, Tanzania, who survived life-threatening obstetric complications. Their narratives will increase understanding of the individual and community-level burden masked behind the statistics of maternal morbidity and mortality in Tanzania. In line with a recent systematic review of women-centred, qualitative maternal morbidity research, this study will contribute to guidance of local and global maternal health agendas.

**Methods:**

This two-phased qualitative study was conducted in July-August 2017 and July-August 2018, and involved three key informants, who were recruited from a maternal near-miss cohort in May 2017 in Mnazi Mmoja Hospital, Zanzibar. The used methods were participant observation, interviews (informal, unstructured and semi-structured), participatory methods and focus group discussions. Data analysis relied primarily on grounded theory, leading to a theoretical model, which was validated repeatedly by the informants and within the study team. The findings were then positioned in the existing literature. Approval was granted by Zanzibar’s Medical Ethical Research Committee (reference number: ZAMREC/0002/JUN/17).

**Results:**

The impact of severe maternal morbidity was found to be multi-dimensional and to extend beyond hospital discharge and thus institutionalized care. Four key areas impacted by maternal morbidities emerged, namely (1) social, (2) sexual and reproductive, (3) psychological, and (4) economic well-being.

**Conclusions:**

This study showed how three women’s lives and livelihoods were profoundly impacted by the severe obstetric complications they had survived, even up to 16 months later. These impacts took a toll on their physical, social, economic, sexual and psychological well-being, and affected family and community members alike. These findings advocate for a holistic, dignified, patient value-based approach to the necessary improvement of maternal health care in low-income settings. Furthermore, it emphasizes the need for strategies to be directed not only towards quality of care during pregnancy and delivery, but also towards support after obstetric complications.

## Background

In women’s health research and policy making, maternal mortality has long been a main indicator of quality of care [1]. In the past decades, this focus has extended to include severe maternal morbidity, acknowledging that maternal health and well-being go beyond survival or death [2, 3]. Assessment of cases of severe maternal morbidity yields epidemiological information, which can be enriched by individual case data, based on clinical audits and patient interviews [4].

Integration of patients’ views in the field of (global) maternal health research has improved understanding of the individual, communal and societal burden of maternal morbidity. Previous research has shown its association with poor physical health, affected sexual functioning, lower quality of life and psychological distress [5–12]. Severe obstetric complications also affect the stability of a household economy and a woman’s social status [7, 8, 13–15]. Much of these findings are universal but variations do exist across different settings and cultures.

The local health care system is pivotal to ensuring people’s health and well-being, but in low-income countries, it is often relatively weak, leaving people out of reach and underserved. Arguably, women who live in poverty in a low-income country are among the most afflicted by poor health care systems, with a high risk of potentially fatal complications during pregnancy and around childbirth.

This study aims to explore the trajectories of three women from Zanzibar, Tanzania, who survived life-threatening obstetric complications, to better understand the statistics of maternal morbidity and mortality, and ultimately guide the locoregional maternal health agenda and improve outcomes.

## Methods

### Research design

A two-phased qualitative study was conducted in July-August 2017 and July-August 2018, drawing on ethnographic research methods. Pairs of research assistants consisting of one Liberal Arts & Sciences student from the Netherlands (mainly responsible for data collection), and one social science student from Zanzibar (mainly responsible for mediation and translation), were formed and matched with a key informant. Prospective selection of the key informants was done in May 2017 in the Obstetrics & Gynaecology department of Mnazi Mmoja Hospital (MMH), Zanzibar. They were selected from a cohort of maternal near-misses that had been identified through setting-adjusted World Health Organization (WHO)’s near-miss criteria [16]. Maternal near-miss (MNM) is defined as life-threatening complications during pregnancy labour or up to 42 days after the end of pregnancy [3]. Selection was based on date of hospital admission (May 2017), residence (within one hour of MMH by public transport) and variety of clinical diagnoses. Data collection was performed during the course of five weeks in both phases: three to four months and 15 to 16 months after occurrence of the near-miss complication, respectively.

### Research setting

The study took place on Unguja, the main island of Zanzibar, an East African archipelago in the Indian Ocean. Zanzibar has a population of approximately 1.3 million people, mainly Kiswahili speakers [17]. Ninety seven percent of inhabitants identify themselves as Muslim, with the remaining 3% identifying as Christian, Sikh and Hindu. Zanzibar is a semi-autonomous region of the United Republic of Tanzania, having a degree of self-governance, including its own Ministry of Health and Social Welfare [18]. Although 80% of Zanzibar’s population resides within five kilometers of a healthcare facility, most of those are understaffed and lack resources [19]. Tanzania’s national fertility rate is 5.2 pregnancies per woman, with a large difference between rural (6.0) and urban (3.8) women, non-formally educated (6.9) and formally educated (3.6) women, and women in the lowest wealth quintile (7.5) and women in the highest wealth quintile (3.1) [18]. Mnazi Mmoja Hospital is the government-run referral hospital, located in Stone Town in the urban-west district. Like other departments, the Obstetrics & Gynaecology department, facilitating around 12,000 deliveries annually, has been struggling with understaffing and insufficient resources. The quality of maternal health care is poor, indicated by a high maternal mortality rate of 401 per 100,000 deliveries [20]. For every maternal death in MMH, at least two women will suffer from near-miss complications but survive [20]. The research team followed three such women in their homes, across different rural locations, all within half an hour drive of MMH.

### Study participants

The key informants were three women, whose fictitious names are Naira, Tatu, and Mada. At the time of her obstetric complication, Naira, aged 32, was married with two healthy children, an eight-year old daughter and a five-year old son. While pregnant, she had not gone to the antenatal care clinic. At three months, she developed severe abdominal pain, after which she lost consciousness. She was brought to the hospital directly by family members where she arrived in a state of hypovolaemic shock and was diagnosed with a ruptured ectopic pregnancy. She underwent an emergency laparotomy during which the fallopian tube, containing the ectopic pregnancy, was removed. Naira was discharged after one week and told to come back for an ultrasound to check if she did not have a pregnancy inside the uterus as well. When she returned to the hospital, she was told she would not get an ultrasound for free, after which she returned home, not knowing whether or not she was still pregnant, and unable to afford an answer.

Tatu, aged 29 years old, had been married with no children. After an uncomplicated pregnancy, she went to the referral hospital to deliver. She underwent a caesarean section due to poor progress of labour, helping her deliver a baby boy of 3.8 kilograms with a good start. For unclear reasons, due to lack of documentation, the baby was sent to the neonatal unit after the delivery. On the third day after delivery, the baby died. Tatu and her husband only met their son after he had passed away. In the next days, Tatu developed a wound infection leading to an emergency hysterectomy on the seventh day after the delivery.

Mada, aged 27, had been married and had six healthy children. She had had two abortions and thus this pregnancy was her ninth. She did not attend the antenatal care clinic. The beginning of her pregnancy was uncomplicated besides slight pains in her legs and stomach and troubles with breathing. At a later stage, she experienced exhaustion and hallucinations which intensified with time. Late at night, she stood up to go to the toilet, had a sudden sensation of freezing and fell on the floor. Her husband had saved up money in case of an emergency, which was used by her brother to buy fuel and bring Mada to the referral hospital. Here, she was diagnosed with severe anaemia and pregnancy-induced hypertension and had a respiratory rate of above 40 breaths per minute on admission. She then received the first of multiple blood transfusions. She was admitted for seven days, feeling terribly sick and confused throughout. Eventually, labour started spontaneously and she gave birth, vaginally, to a healthy baby boy of 4.2 kilograms. After delivery, she received another blood transfusion.

Next to them, members of these women’s households, extended family members and wider social circles participated in this study. Inclusion criteria for these informants were age of at least 18 years old, willingness to participate and close relation the key informant.

### Data collection methods

Each participant’s household was visited three to five days per week by one of the research pairs. Participant observation was the main method used, allowing the researchers to familiarize themselves with the study participants, household members and the family structures. This was stimulated by regular and frequent full day presence of the researchers, fostering a trusted relationship between the researchers and the participants whilst allowing for close observation of household and community dynamics. The main focus was placed on the woman’s current wellbeing and its relation to the complication experienced during her pregnancy.

One-off interviews and repeat interviews took place with the key participants, household members, relatives and people from their inner social circle. Three different styles of interviewing were used: informal, unstructured and semi-structured interviews.

Participatory methods were used during interviews as data collections and mapping tools, such as Venn diagrams to map dynamic social relations and pie charts to visualize household spending and earnings.

Four focus group discussions (FGD) were held in 2017, one with each family, with varying numbers of participants. In 2018, there were two interactive FGD-like meetings, which will be further referred to as FGDs as well, though we recognize that the official definition of FGD includes at least five participants [21]. One of these FGDs consisted of the three key informants and another was with three male relations, Mada’s husband and Naira’s father and brother. Each FGD took one to two hours and took place at the participants’ residencies in 2017 and in a neutral space in Zanzibar City in 2018. A participatory method utilized during the FGD was the problem tree, with the maternal near-miss morbidity presented as the core problem. During all FGDs, mediated discussion took place, with notes taken by a Kiswahili- and English-speaking researcher, and cross-checking of the English translation by a second researcher. Subsequently, the two researchers brought up follow-up questions.

### Collection and storage

Field notes were taken throughout the study and saved as jottings in notebooks in hard copy and backed up later as coherent larger field notes in a shared digital database. Photographs and audio-records were taken if deemed of additional value and were then saved digitally. Daily and weekly inter-researcher discussions were held during the two study periods, including weekly discussions with TH and monthly discussions with the team’s anthropologists.

### Analysis

The combination of various methods of data collections, various types of data and multiple observers allowed for triangulation of the findings, increasing validity of data and decreasing the risk of observer bias [22]. Data analysis relied mainly on grounded theory, an anthropological technique to process qualitative data. From this, we constructed a dynamic theoretical model, which was strengthened through daily inter-colleague discussions, after which it was applied the following study day. Thus, it was finetuned continuously and validated repeatedly with the informants as well as within the study team. The concept of grounded theory is based on inductive, open coding and is focused on first gathering data and then examining what conclusions arise from this data. Notes and transcripts from the focus group discussions, interviews and participant observations were read and re-read, after which a content analysis was performed. This entailed an initial stage of axial coding, in which various categories were delineated and described to then be evaluated based on their relevance. Through coding the collected data, the relationships between the different categories and subcategories were investigated and clarified. This stage was followed by a stage of selective coding, which included the determination of the most important categories and concepts, studying the relationships between these concepts and verifying them, and finally, formulating our definitive theoretical model of which the findings were positioned in the existing literature [23].

### Ethical considerations

The study protocol was approved by Zanzibar’s Medical Ethical Research Committee (reference number: ZAMREC/0002/JUN/17). Accordingly, confidentiality was guaranteed during data entry, processing, and storage. The study participants were given pseudonyms. Written or, in the case of illiteracy, verbal informed consent was obtained from all participants, primarily of the three main participants and heads of the respective households. In case of psychological or psychiatric crises, the participants would be referred to the psychiatric out-patient clinic.

## Results

Four main areas of relevance in the daily lives of the key informants were identified: social sphere, sexual and reproductive well-being, psychological well-being and economic status.

### Impacts in the social sphere

A significant impact on the key participants’ social structures was observed. While in the hospital, the three key informants relied greatly on their relatives for practical help (e.g. going to the bathroom and getting medicine), as well as for emotional support (e.g. calming them down, giving them hope). About this, Tatu’s mother said: *“I slept on the hospital benches for two months to take care of my disabled child.”*

Once back home, the women were reportedly more dependent on their social networks for support than before the near-miss: the physical inability to resume daily activities, such as household tasks and childcare, led to a long-term change in of the women’s social spheres. In the cases of Tatu and Mada, one or more family members moved in with them temporarily, to assist them in daily life. On this, Mada’s mother in law said: *“We only have one rule in this house: we do everything together; we cooperate”*. Naira moved in with her mother for three months, after which she moved back to her own house, concerned she was a strain on her mother’s household resources.

All key informants described the emotional support they received from their families as essential. Naira said: “*they offered kind words and advice, reminding me I am strong”*. Tatu described her husband’s role in her recovery, taking time off work to be with her, about which he said: *“I have accepted it, I needed to. I need to be strong to support her”*.

Various family members mentioned how they encouraged and prayed for their recovering relative to accept their situation, spiritually guided by the principle of God’s will, as Tatu’s aunt said: *“We give her hope, saying: this is all God’s plan”.*

All three women initially experienced social isolation, about which Naira’s mother said: *“She does not have the ability to go around and visit friends and family anymore”*. In the second study phase, this seemed to have cleared up. Naira mentioned to now have conversations with people about her complications, finding that beneficial. All key informants said to not find major implications on their social environments. Mada’s husband Jul added: *“Now there is no problem, life is the same as always”*.

### Impacts on sexual and reproductive well-being

The lack of information or knowledge regarding the consequences of the complications they had gave rise to fear of recurrence and insecurities around sexuality and reproductive capacity. Tatu underwent a hysterectomy for which she had not been able to be informed nor give consent. Though this is an extreme example, Naira and Mada similarly received little counselling on their sexual and reproductive health following the complication. Naira expressed her concerns: *“If you get any complication, especially related to the reproductive system, you have a worry you will not have the same ability and management of sex”.* In the hospital, she had not been counseled and, consequently, waited for almost a year after the complication before becoming sexually active again about which she said: *“I have a lot of stress. I worry that if I have another pregnancy, I will get more problems”*. Naira and Mada also mentioned that they were not informed of possible effects on their menstrual cycle but both experienced post-complication disruptions of their menstrual cycle, with Naira in the second study phase still experiencing pain during her short and irregular menstruations.

However, such impacts were fading in the second study phase and the participants reported that their sexual lives were returning to normal. Both Mada and Naira considered to start using contraception. Naira said she wants to use condoms to reduce the chance of pregnancy, and asked questions during the research to broaden her own knowledge about reproductive health. Mada originally contemplated a hysterectomy to prevent a future pregnancy, but she decided against this in the second year after the complication when she expressed her interest in contraceptives, whilst keeping concerns about possible side effects. During the second study phase, she was using *shakhe* as a contraceptive, a method of spiritual contraception involving water blessed by a religious leader reading the Quran. Following the hysterectomy, Tatu is no longer able to have children, but as her mother said: “*the complication has not affected her will to be a mother*”. Tatu and her husband adopted her sister’s son, who refers to her as ‘mama’. In addition, her husband plans on taking another wife, who can then bear children to make the family bigger. None of them indicated lasting effects of the complication on their sexual pleasure, as Tatu said: *“nothing has changed in the way I am with my husband”.*

### Impacts on economic status

The three women described their socio-economic status as low, with their financial situations heavily affected by the sickness, the costs of hospital admission, post-admission costs for official and traditional treatments and their loss of ability to work. Women and their husbands described how spending and saving patterns had been adjusted, with expected future effects. Various members of the three households described increases of health care expenses from the onset of the complication onwards, lasting throughout the second year. Naira and Mada both estimated a five percent increase in expenses, and Tatu’s increase was estimated to be even higher as she had sought medical care in a private hospital. In addition to hospital care, Naira and Mada sought alleviation through paid traditional and spiritual medicine services. Exemplary of this are *kombe*, sessions with a spiritual healer and herbal treatments, considered to be relatively costly. Other major financial burdens that were mentioned were transportation to and from the hospital and the purchase of healing foods, to support recovery.

In pursuance of *sadaqah*, one of the five pillars of Islam meaning ‘voluntary charity’ and ‘righteousness’, many relatives, neighbours and friends were said to have contributed financially to the health care expenses. Nevertheless, some of these contributions were loans.

Directly caused by their affected physical health, Naira and Tatu reported a decrease in their ability to work, with a consequent lower household income. Naira, who, before the complication worked daily on her parents’ farm and ran her own juice shop, went back to farming nine months after the near-miss, but still felt constrained: “*Before, I could harvest two clove trees in one day, but now, I barely do even one*”. She had to ask her brother and sister for assistance in her juice business, and thus had to share the profit with them. In addition, Naira’s husband Othman mentioned how he had to work more to compensate, leading him to be more stressed “*because of life”* and not being able to spend as much time with his friends and family. Tatu’s husband in the first study phase reported similar findings. Luckily, when Tatu went back to part-time work after nine months, she continued to receive a full-time salary.

For Mada, her sickness placed more responsibility on her husband and other family members, like her sister, her mother and her mother-in-law, who temporarily took over her household work and taking care of the family. Because of ongoing blood loss, Mada was also not able to go to the market, make and sell juice and collect and sell wood during the first months after the complication. Fifteen months after the complication, she proudly said to be recovered enough physically to be as productive as she used to be. Her husband Jul, though, was still experiencing an increased pressure to provide for the family, because of the increase in medical costs, their new child, a failed business investment and depleted savings. Consequently, he said: “*I cannot afford to take a day off*”. He describes his job as a brick-maker as physically hard and uncertain, as the cement that is required for the making of bricks is becoming increasingly more difficult to obtain. This all worries him greatly, feeling it is a husband’s duty to take care of the family financially so that his children and wife can have a better future. He furthermore explained how he used to be part of a savings collective of nine men who all regularly put in a predetermined amount of money (20,000 Tanzanian shillings per day, equivalent to around 10 United States dollars, in Jul’s case), the sum of which then could be used by a different member each month. However, as he is currently unable to save anything, he can no longer be part of the collective that helped him pay for his wife’s treatment. For Tatu and her husband, saving has also become more difficult. Having exhausted their savings by paying for medication and treatment and still experiencing the financial consequences of Tatu’s compromised health, they both feel the pressure to save as much money as possible. Nevertheless, both mention that they have not had the opportunity to save more than half of what they used to.

Lifetime-earning prospects were also affected by the increase in spending on care, implying that the financial burden for the families will extend to the future as well. For example, Jul’s plans to set up a small restaurant in which Mada then can sell homemade *urojo*, a local soup, were compromised because of last-minute overbidding that caused him to lose the venue and made the purchases inventory redundant, something which probably would not have happened if the money spent on Mada’s treatment would have still been available. “*If we had the shop, life would be good now; less stress*”, he said, referring to him having to resume his physically intensive work as brickmaker and his wife not having the chance to be more independent financially. In line with this, the felt financial burden led Naira to postpone seeking of care for her problematic menstruation. As she said she could not afford the treatment and medication she would need and, thus, had to accept her current physical disabilities.

An important theme that emerged is how the decrease in economic productivity led to a decrease in the women’s agency. To varying degrees, the key participants reported an increase in financial dependency on their husband, e.g. for medication and healing foods and other commodities they were used to buy by themselves. They kept looking for ways to increase their (household) income, through making and selling juice for example. Nevertheless, longer working hours by the husbands, receiving financial support from their social network and sharing living spaces, clothes and food were in all three situations needed for the households to continue to provide their members with all life’s essentials.

### Impacts on psychological well-being

Key- and other participants shared that the maternal near-miss event had serious implications on the psychological wellbeing of patients, though widely varying in form and magnitude. The factors that were mentioned to influence the level of psychological impact most were the women’s coping mechanisms, the type and level of physical (dis)abilities and the type(s) of loss they had experienced.

All key participants experienced profound fear and stress during and after the complication. Mada described this as *death anxiety*, referring to the time when she had to rush to the hospital and had woken up her children to say her *“final goodbyes*” in the middle of the night. Tatu mentioned uncertainty and poor communication as the causes of stress and anxiety she experienced during hospitalization. This is in line with Mada’s comment about her doctor’s unsubstantiated announcement of her needing to get a caesarean section, a procedure which she believed had led to the death of two of her family members in the past. With Mada’s blood pressure rising dangerously, her mother and mother-in-law, who were with her in the hospital at the time, were terrified, as Mada’s mother-in-law explained: “*That is why we [all] got so emotional when we heard the doctor say Mada needed a caesarean; we thought this was the main reason for dying*”. Nevertheless, her mother and mother-in-law did not show this fear to Mada and supported her with words like “*Allah will help you deliver a healthy baby*”. Naira left the hospital not knowing whether she was still pregnant, which led to a clear disruption of feelings of bodily integrity. Once back home, Mada continued to have hallucinations and nightmares, as she had had the month prior to the hospitalization. At times, these were so disturbing that she would not be able to sleep at night, driving her to exhaustion and health deterioration. The participants described this as *mjusi*, an evil ghost that takes possession of a person during bleeding. Naira also suffered from this, leading to strong anxiety and worry. They both underwent treatment for *mjusi* by *kombe*, a mixture of Quran verses written on paper, boiled in water and drank this several times a day. They expected improvement over time, as *mjusi* is expected to disappear once the bleeding has stopped.

Psychological as well as physical phenomena were attributed to Allah’s will as “*he is in control of everything*” and “*is everything there is*”, as Mada phrased it. Therefore, religion was found to be the most important coping strategy of the key participants. As they explained, *tawakkul*, or *supra*, is one of the core elements of “*being a good Muslim*”, standing for trusting in God’s plan and acceptance of whatever comes on the path of life. All interviewees expressed this concept in understanding the complications and their consequences, finding the question of why to be redundant as it is impossible to know and change what God has planned. Religion was further mentioned to help overcome frustration, regain hope and trust, and to provide a general sense of comfort. In Naira’s words: “*all problems belong to God, and it is in his hands to alleviate the problems and offer solutions*”.

The key informants had their own ways of further dealing with distress. Mada expressed to be more introvert, claiming that in moments of distress *“I talk to my soul”*, whilst Naira and Tatu said they shared their emotions more with friends and family.

With the eye on the future, the key informants expressed that their complicated delivery could be a “*lesson for others*” and that “*you will just have to accept it and deal with it”*. Mada and her relatives mentioned that besides religion, the fact that she had a healthy baby helped in “*forgetting the past*”, “*living life as always*” and “*looking at the future*”. Naira explained that further strengthening of her psychological well-being was because of regained sexuality, as being sexually active with her husband made her feel “*like a woman again*”. This appeared to promote her sense of autonomy and helped her to let go of past events. Life as it was before the complication took further shape with starting to work again. Tatu explained to have regained her independence once she had gone back to work: “*I feel like I am as independent now as I was before*”. Mada shared that being able to take care of the household and the children herself again made her feel like she had regained agency.

## Discussion

This study has displayed the trajectories of three Zanzibari women who survived a near-miss complication in their pregnancy. These trajectories were outlined trough narratives of the women themselves and of people close to them, as well as through observations from the researchers. The study aimed to explore how such complications, three to four months and 15 to 16 months after, impacted the women’s daily lives and their households’ daily structures within the specific local social and cultural context. The findings have shown how multi-dimensional the impact of severe maternal morbidity is, and how it extends beyond hospital discharge, at which point institutionalized care usually stops.

Four main areas of relevance have emerged: the social sphere, sexual and reproductive well-being, economic status and psychological well-being. During the first few months, physical complaints restricted women’s functioning in their household and beyond. Consequently, in-house family structures changed temporarily, with the women being dependent on kin for help. Struggling initially with a certain degree of social isolation, effects on the social life had mostly diminished in the second study phase, 15 to 16 months after the complication.

In terms of sexual and reproductive well-being, the factor of time also alleviated concerns and complaints. In the first few months, however, worries and a lack of biomedical understanding burdened all women heavily. This was aggravated because of poor medical counselling, loss of reproductive capacity after a (life-saving) hysterectomy, constrained accessibility to health care services, limited understanding of (patho)physiology of the reproductive organs and misinformation on contraceptives.

Immediate costs around the complications as well as long-term increases in health-related expenses economically burdened not only the women, their husbands and their household but reached to their more distant relatives and acquaintances. Our informants sought health care in the official as well as the traditional and spiritual sector, with the latter being a non-neglectable source of costs. Next to medical expenses, the main reason for economic stress was the women’s (temporary) loss of income in two of the cases, since they had been self-employed and physically constrained to perform their income-generating work.

Psychological distress seemed to be intertwined with all the above. The women as well as their husbands and parents suffered most in the first months after the near-miss event. When they had regained physical strength, re-integrated in their households and communities and started income-generating work, all three women expressed a sense of recovery and agency.

### In the context of existing literature

#### Social isolation

In the aftermath of severe obstetric complications, other authors similarly described effects in the social, psychological, economic and reproductive health spheres. The experience of social isolation once back home was equally described by British women, who survived a maternal near-miss event, due to emotional and physical restrictions [8]. In Burkina Faso [24], women also experienced social destabilization and insecurity around their social identity as in this study’s setting in Zanzibar.

#### Affected sexual and reproductive health

Social expectations, lack of information and knowledge and women’s caution dictate the near-miss survivors’ sexual and reproductive well-being in the aftermath. As in our study, in West-African low-income settings [5, 6] as well as in high-income settings [25, 26], women struggle with desires and decisions around their fertility and future pregnancies. A uniquely harsh burden is upon those who have lost their biological ability to conceive [26], as we observed in one of this study’s key participants who underwent an emergency hysterectomy. For the others, determining if and when to start sexual activity and family planning practices, is much dictated by the course of the near-miss event. As described in Burkina Faso, this is strongly influenced by the outcome of the near-miss pregnancy [27].

#### The financial burden

There is sparse literature that specifically looks into economic consequences of health crises. Similar to this study, Storeng et al. described the struggles with finding money to cover health care expenses in Burkina Faso, underlining the economic shock caused by the near-miss event, with economic as well as social consequences [13]. In the same Burkina Faso study population, the association between poverty and the risk of severe obstetric complications [28], the affected household economy [24] and the women’s loss of income-generating activities [29] are strong arguments for policy change for maternal health care improvement. Equally so, in our study setting, the financial burden ranged beyond the acute in-hospital phase, involving kin and acquaintances and giving up business ambitions to make ends meet. With a growing body of work on (severe) maternal morbidity and with suggested strong links between poverty and poor health outcomes, as shown in economic studies on maternal mortality [30–35], the nexus between economic resources and maternal morbidity deserves to be assessed.

#### Resilience

Following Obrist’s definition of resilience as “proactive capacities to anticipate change and search for new opportunities” [36], we observed exactly that in our key participants and their households. Nevertheless, in the first study phase the distress was high in many dimensions, and there seems to be a potential role for the medical care system to alleviate that somehow. As found in a British maternal near-miss study [37], this study’s key participants suffered from the lack of counselling during hospital admission, not or incompletely understanding what they had gone through and what to expect in the aftermath. Early postpartum care to avert mental distress, for example postpartum depression has been proposed in studies from Benin [38] as well as from Britain [8]. The Benin study specifically mentions the maternal near-miss population and those women that experienced perinatal death to be targeted with preventative and diagnostic strategies [39]. Hinton et al. make a case for the potential role for primary health care structures in this, with another British study [40] additionally advocating for emotional support mechanisms for the partners of the women.

#### Implications for research and practice

This study advocates for a transformation of how we address pregnancy- and childbirth-related care globally, putting the patient and her value at the core of research and practice. Our standpoint is in line with that of a recent systematic thematic review of qualitative literature reporting experiences and perspectives of women around the world, who have gone through maternal morbidity, to perform high-quality, broad and women-centered research and let this guide our (global) health agendas [40]. Our study shows that in poor communities, despite effective coping strategies and resilience at individual and community level, women are at a high risk of long-lasting impacts of maternal complications, due to limited financial resources and access to knowledge and care. Strategies to reduce the burden of maternal complications need to be directed not only towards quality of care during pregnancy and delivery but to provide support after life-threatening complications. They should also recognize the importance of supporting economic independence and agency of the women concerned. We advocate for setting-specific biomedical, medical anthropological, psychological and economic studies guided by patients’ reports. This is along the line of the concept of value-based health-care, which defines patient value as patient-relevant outcomes in relation to costs per patient to achieve those outcomes [41].

#### Strengths and limitations

This study is among the first to be found in ethnographic literature, focusing on women who have survived life-threatening obstetric complications. It is also the first to do so in Zanzibar, Tanzania. In trying to unravel the meaning of compromised maternal health in this socio-cultural setting, we inevitably found universal concepts, applicable to persons who suffered from similar conditions though living elsewhere, as well as to persons who suffered from different conditions but live in similar environments as our informants. Nevertheless, due to the specifics of the study setting and the relatively low number of participants, findings might not apply to women and households in Zanzibar residing in other districts or having other socioeconomic statuses. Additionally, the three key informants had been selected in the region’s referral hospital, which likely caused selection bias. Within Zanzibar’s health care referral system, however, we expect that those in our target population - the women who are most severely ill but survive - most likely find care in the referral hospital at one point during their illness.

The main strengths of this study are its longitudinal character and the two-fold study phase, in two consecutive years. This enabled not only to witness changes over time, up until 16 months after the near-miss event, but also to build on findings from the first year and perform in-depth explorations of previously touched upon topics during the second year. This added to the study’s detailed character.

The study team was varied in terms of personal and professional backgrounds and experience. We acknowledge how this might have challenged optimal execution and analysis of the research, although we feel it mostly enriched the study, supported by regular and frequent inter-researcher discussions.

## Conclusions

This study displayed three unique whilst, simultaneously, universally applicable stories. Our key informants were three women, from challenging socioeconomic backgrounds, who survived severe obstetric complications but had to deal with large and far-reaching effects in the aftermath of the physical illness. This study showed how this impact, up to 16 months after the complications, goes beyond the acute in-hospital phase and beyond the physical health status, affecting social, economic, sexual and psychological spheres, involving family and community members. Enriching epidemiological outcomes, this study advocates for a holistic, patient value-based approach to much-desired improvement of antenatal, perinatal and postpartum maternal health care.

## Data Availability

The datasets generated and analysed during the current study are available from the corresponding author on reasonable request.

## Abbreviations

FGD: focus group discussion
MMH: Mnazi Mmoja Hospital
MNM: maternal near-miss
WHO: World Health Organization
